# 

**Published:** 1876-06

**Authors:** 


### *California State Dental Association.*

Dear Sir :—In accordance with the custom of my predecessors, I hereby announce the Seventh Annual Session of our Association, which will convene in San Francisco, Tuesday, June 15th, at 10 o'clock A. M., and will continue four days.

A most cordial invitation is extended to every worthy member of the Profession to be present on that occasion, and all so desiring, if possessing the necessary qualifications, will be as cordially admitted into active membership.

Our very liberal constitution only requires you to have been in practice THREE YEARS, exclusive of your term of pupilage, or the holder of a diploma from a respectable Dental College, and the possessor of a good moral character. Also, that you have your application for membership endorsed by two members of the Association.

Our Code of Dental Ethics, to which all members must subscribe, contains nothing to which any honorable practitioner can object. It originated with the American Dental Association, has been adopted by nearly all the State and local Associations, and approved by all the Dental Colleges.

Our objects are—" The promotion of the highest excellence in the science and art of Dentistry, and its collateral branches; the cultivation of closer professional relations and good-fellowship among our members and, collectively, to represent and have cognizance of the common interests of the Dental Profession."

I trust you will consider it YOUR duty, as it is the duty of every honorable practitioner, to aid in the carrying out of said objects, that our chosen profession may enjoy more of the confidence and respect of our fellow-men, and the sooner acquire a rank second to no honored profession of our land. We are living in an age replete with manifestations of the incontrovertible fact that through associative effort, all professions throughout the world are being more rapidly elevated to their proper sphere, than by any other known means; and, in the near future those who do not exhibit a " progressive spirit" by acquiring and maintaining an active membership in the Associations of their respective professions, will be called upon by an intelligent and appreciative public to " step down and out," that their places may be filled by those of real and not pretended merit.

Please be kind enough to make a careful and impartial examination into all the facts concerning your qualifications as a practitioner. If, fortunately, they are such as to justly entitle you to rank with those who have attained the higher eminences in our profession, I trust you will deem it ALL

THE MORE your duty to cheerfully give us the benefit of your professional knowledge, thereby assisting in the ever laudable effort to elevate all to the highest degree of professional excellence. If, UNFORTUNATELY, you do not possess the requisite qualifications to enable you to do justice to your patients, your profession and yourself, I entreat you, let this Centennial year mark the era of your "departure" for a higher, more exalted sphere of usefulness, with a full determination to maintain in the future a professional standing that will prove you worthy your honorable vocation.

In conclusion, permit me to remind you that "he who looses sight of his profession's character, must soon suffer in his own, and that your profession, if worthy your choice, should receive all your energies, without which you cannot expect success."

Very Respectfully, yours,

H. J. PLOMTEAUX,

*President California State Dental Association.*

---

*The District Dental Society*, of the Seventh Judicial District of the State of New York, will hold its Eighth Annual Meeting at Rochester, N. Y., Tuesday and Wednesday, June 6 and 7, 1876.

The session will commence at 2 o'olock of the first day and continue until the close of the second.

Members of the Profession from other Societies, States and the Dominion, are cordially invited to be present and take an active part in the discussions.

Dentists who reside in the Seventh Judicial District, and who are not Members of the Society, may make application for Membership, previous to the first day of the meeting, to the Chairman of the Board of Censors.

Article Eighth of the By-Laws reads as follows:

Sec. 1. Every person elected to active membership in this Society shall before signing the By-laws and Code of Ethics, pay to the Treasurer an initiation fee of five dollars.

Sec. 2. Every person who shall pass a satisfactory examination before the Board of Censors, and be recommended for a Certificate of Qualification, shall pay the Treasurer the sum of ten dollars before the Certificate is conferred.

Members and others are hereby informed, that a limited number of handsomely bound copies of two years "Proceedings of the Dental Society of the State of New York," have been received by the Recording Secretary, and will be mailed to any address on receipt of 65 cents.

---

### SELECTED ARTICLES.

### TO READERS AND CORRESPONDENTS.

Communications intended for publication, and exchange journals and papers, should be addressed to the EDITOR OF THE AMERICAN JOURNAL OF DENTAL SCIENCE, 259 N. Eutaw St., Baltimore, Md. All letters relating to the business of the journal, or containing cash remittances, to be directed to Messrs. SNOWDEN & COWMAN. Articles for publication should be received by the editor at least three weeks previous to the first day of the month on which the number in which it is designed for them to appear, is issued.

### CONTENTS:

### ORIGINAL COMMUNICATIONS.

### MONTHLY SUMMARY.

☞ The American Journal of Dental Science will contain fifty pages of reading matter in each number, making a volume of not less than

*Six Hundred* pages

EXCLUSIVE OF ADVERTISEMENTS

THE

### AMERICAN JOURNAL OF DENTAL SCIENCE.

**F. J. S. GORGAS, M. D., D. D. S., Editor.**

The *Journal* is issued on the first of each month and contains not less than *fifty* pages of reading matter in each number, exclusive of advertisements, making a volume of *six hundred* pages per annum.

In each number will be found Original Communications, Correspondence, Selected Articles, Monthly Summary, Bibliographical Notices and Editorial Matter, and every effort will be exerted to render the *Journal* worthy of the patronage of the Dental profession.

A generous co-operation is respectfully solicited from the members of the profession; and as the *Journal* has now a printing office of its own, which materially lessens the cost of its publication, it will be issued at the reduced subscription price of

**$2.50 Per Annum in Advance.**

Communications are respectfully solicited on all subjects pertaining to the practice of dentistry, as well as reports of cases occurring in dental practice.

### RATES OF ADVERTISING.

| | 1 MO. | 2 MO. | 3 MO. | 6 MO. | 12 MO. |
|---|---|---|---|---|---|
| 1 PAGE. | $15 00 | $22 50 | $30 00 | $50 00 | $100 00 |
| ½ " | 10 00 | 15 00 | 20 00 | 30 00 | 50 00 |
| ¼ " | 7 00 | 11 00 | 15 00 | 20 00 | 30 00 |
| ⅛ " | 5 00 | 8 00 | 11 00 | 15 00 | 20 00 |

All original communications designed for publication, works for review, new instruments for notice, exchanges, &c., should be directed to the editor, 259 N. Eutaw St., Baltimore, Md.

All advertisements and subscriptions should be addressed to

SNOWDEN & COWMAN,
*Publishers of the Am. Journal of Dental Science.*
86 W. FAYETTE ST. BET. CHARLES & LIBERTY STS.,
BALTIMORE, MD.

